# Multidimensional daily diary of fatigue-fibromyalgia-17 items (MDF-fibro-17): part 2 psychometric evaluation in fibromyalgia patients

**DOI:** 10.1186/s12891-017-1545-x

**Published:** 2017-05-18

**Authors:** Y. Li, S. Morris, J. Cole, S. Dube’, J. A. M. Smith, C. Burbridge, T. Symonds, S. Hudgens, W. Wang

**Affiliations:** 1 Former employees, current consultants of Theravance Biopharma US, Inc, 901 Gateway Boulevard, South San Francisco, CA 94080 USA; 2 Former employees of Covance Market Access, San Diego, CA USA; 3 Consulting Associate Professor, Department of Psychiatry and Behavioral Sciences, Stanford University School of Medicine, Stanford, 94305 CA USA; 4 Adjunct Professor, University of Pittsburgh Schools of Medicine, Pittsburg, 15260 PA USA; 5 Clinical Outcomes Solutions, Unit 68 Basepoint, Shearway Business Park, Shearway Road, Folkestone, Kent UK; 6 Clinical Outcomes Solutions, 1790 E. River Rd., Suite 205, Tucson, AZ 85718 USA; 7 Theravance Biopharma US, Inc., San Francisco, CA USA

**Keywords:** Fibromyalgia, Fatigue, Diary, Patient reported outcome (PRO), Psychometrics, Confirmatory factor analysis (CFA), Validity, Reliability, responder, MDF-Fibro-17

## Abstract

**Background:**

The Multidimensional Daily Diary of Fatigue-Fibromyalgia-17 instrument (MDF-Fibro-17) has been developed for use in fibromyalgia (FM) clinical studies and includes 5 domains: Global Fatigue Experience, Cognitive Fatigue, Physical Fatigue, Motivation, and Impact on Function. Psychometric properties of the MDF-Fibro-17 needed to demonstrate the appropriateness of using this instrument in clinical studies are presented.

**Methods:**

Psychometric analyses were conducted to evaluate the factor structure, reliability, validity, and responsiveness of the MDF-Fibro-17 using data from a Phase 2 clinical study of FM patients (*N* = 381). Confirmatory factor analyses (CFA) were performed to ensure understanding of the multidimensional domain structure, and a secondary factor analysis of the domains examined the appropriateness of calculating a total score in addition to domain scores. Longitudinal psychometric analyses (test-retest reliability and responder analysis) were also conducted on the data from Baseline to Week 6.

**Results:**

The CFA supported the 17-item, 5 domain structure of this instrument as the best fit of the data: comparative fit index (CFI) and non-normed fit index (NNFI) were 0.997 and 0.992 respectively, standardized root mean square residual (SRMR) was 0.010 and the root mean square error of approximation (RMSEA) was 0.06. In addition, total score (CFI and NNFI both 0.95) met required standards. For the total and 5 domain scores, reliability and validity data were acceptable: test-retest and internal consistency were above 0.9; correlations were as expected with the Global Fatigue Index (GFI) (0.62-0.75), Fibromyalgia Impact Questionnaire (FIQ) Total (0.59–0.71), and 36-Item Short Form Health Survey (SF-36) vitality (VT) (0.43–0.53); and discrimination was shown using quintile scores for the GFI, FIQ Total, and Pain Numeric Rating Scale (NRS) quartiles. In addition, sensitivity to change was demonstrated with an overall mean responder score of -2.59 using anchor-based methods.

**Conclusion:**

The MDF-Fibro-17 reliably measures 5 domains of FM-related fatigue and psychometric evaluation confirms that this measure meets or exceeds each of the predefined acceptable thresholds for evidence of reliability, validity, and responsiveness to changes in clinical status. This suggests that the MDF-Fibro-17 is an appropriate and responsive measure of FM-related fatigue in clinical studies.

## Background

Fibromyalgia (FM) is a disorder characterized by chronic widespread pain and tenderness that is estimated to affect 0.5–10% of the worldwide population, with approximately 2–3% (greater than 5 million individuals) of the affected individuals present in the United States (US) alone [1–5]. Patients with FM often experience other symptoms, such as fatigue, impaired sleep, negative mood, cognitive limitations, and physical functioning limitations, leading to a reduced health-related quality of life (HRQoL) [6, 7]. Beyond pain, fatigue is commonly identified as one of the most bothersome and disabling symptoms, reported by greater than 80% of FM patients [1, 5, 8]. Patients often describe fatigue as “disruptive or extremely disruptive” to their HRQoL [9].

There is a growing body of evidence from both clinical and regulatory communities supporting FM-related fatigue as a multidimensional concept [1, 9–11]. Additional research on this phenomenon is needed within the context of clinical studies to fully understand the dimensionality as well as ascertain the ability of a single measure to saturate the construct of fatigue. The Multidimensional Daily Diary of Fatigue—Fibromyalgia-17 items (MDF-Fibro-17) is being developed for this purpose; to allow for the exploration and assessment of different components of FM-related fatigue (cognitive versus physical, etc.) in clinical trials while capturing the overall complexity of this experience [12].

Existing research that had been conducted with FM patients for concept elicitation [1], cognitive debriefing and the pilot testing [9] of an initial pool of 23 items was reviewed and used to inform the development of a multidimensional assessment of FM-related fatigue [12]. Five dimensions were identified to reflect the broad experience of FM-related fatigue: Global Fatigue Experience, Cognitive Fatigue, Physical Fatigue, Motivation, and Impact on Function. Qualitative and quantitative item-level evaluation suggested that 17 of the original pool of 23 items best supported the conceptual model. This resulted in the 17 item MDF-Fibro-17 being proposed [12].

The original qualitative work confirmed the content validity of the instrument, [12] developed for use in FM clinical studies in accordance with the Food and Drug Administration (FDA) guidance for patient reported outcome (PRO) development [13]. Further work however was needed to conduct psychometric analyses to support the appropriateness of the MDF-Fibro-17 for use in FM clinical studies. The original 23 item pool were therefore administered in a Phase 2 clinical study of TD-9855 (NCT01693692), and psychometric analyses were conducted and are presented in this article. The Phase 2 clinical study (NCT01693692) was a randomized, double-blind, parallel group, placebo controlled study conducted to investigate whether an investigative product, TD-9855, was effective in treating patients with fibromyalgia. TD-9855 is a potent reuptake inhibitor with modest selectivity for inhibition of norepinephrine reuptake and good central nervous system penetration properties in humans. It was hypothesized that TD-9855 would offer the potential for robust pain relief while minimizing any putative serotonergic side effects such as nausea, somnolence, fatigue, and sexual dysfunction. In addition, the majority of fibromyalgia patients suffer comorbid fatigue, therefore reduction in serotonergic activity could be beneficial [14]. Based on this, the primary endpoint for this study was fibromyalgia pain and the exploratory endpoint was fibromyalgia-related fatigue. The Multidimensional Assessment of Fatigue (MAF) was included in the study along with the 23-item pool used to develop the MDF-Fibro-17. The study included 392 subjects treated with TD-9855 2 dose levels or placebo with a ratio of approximately 2 to 1. This quantitative analysis was conducted to confirm whether the MDF-Fibro-17 is an acceptable instrument for the measurement of FM-related fatigue in clinical trials in adult patients with FM, and includes parameters associated with the reliability and validity of the individual items and scores of the MDF-Fibro-17 as well as the responsiveness and hence, interpretability of the measure.

## Methods

The original pool of 23 items developed from the qualitative work was incorporated into a Phase 2 study of TD-9855, an investigational norepinephrine and serotonin reuptake inhibitor, in patients with FM [15]. Patients were required to be diagnosed with FM according to the 1990 American College of Rheumatology criteria,[3] be aged 18–65 years, and to have a self-reported pain level of at least 4 on an 11-point Numeric Rating Scale (NRS). Each subject signed an Institutional Review Board or Independent Ethics Committee approved informed consent form prior to participating in this study. Ethical approval for the original qualitative research was provided by Copernicus, a US centralized Independent Review Board. Ethical approval for the Pfizer cross-sectional validation study was provided by the Schulman Associates Institutional Review Board, Inc. and the University of Cincinnati Institutional Review Board. Ethical approval was obtained for the Theravance validation study at a site level, with each site obtaining approval individually. The 23 items were programmed onto a personal digital assistant (PDA) hand-held electronic device, to be completed by the patients at the end of each day during the placebo run-in period (Days -7 to -1), the treatment period (Days 1 to 43), and the post-treatment washout period (Days 44 to 57). Training for investigators and patients in the use of the PDA and completion of the diary in accordance with study procedures was provided in addition to a quick reference guide.

Patients were instructed to complete all items at approximately the same time every evening, and a restricted time-window for completion was programmed between the hours of 17:00 and 24:00. Retrospective completion of missed days was not allowed. The diary questions were presented sequentially and the option to skip items was not provided.

Each item was presented as a 0–10 NRS anchored by “not at all” at 0 and “extremely” at 10; higher scores indicated greater fatigue severity for 22 of the 23 items. A weekly score was calculated as the mean of the available data if greater than 4 entries were completed within the 7-day period. Observations less than 4 entries were considered missing with no imputation. All items were evaluated on an item level to confirm the hypothesized 5 domain, and a 17-item fit of the data to the conceptual model identified previously in qualitative work [12]. The 5 domain scores (Global Fatigue Experience, Cognitive Fatigue, Physical Fatigue, Motivation, and Impact on Function) were calculated as the summed average of item scores in each domain. A total score was calculated as the average of the domain scores (also ranging from 0 to 10).

A number of additional instruments were included in the study and used to inform the psychometric evaluation of the MDF-Fibro-17 (see Table 1 for further details.)

**Table 1:** Instruments used to inform the psychometric evaluation of the MDF-Fibro-17

Measure	Concepts evaluated
Multi-dimensional Assessment of Fatigue (MAF) [42]	Global Fatigue Index: severity; distress; degree of interference in activities of daily living; timing (PRO)
Medical Outcomes Study 36 item Short-Form Health Survey Version 2 (SF-36) [43]	HRQoL: Physical function; Role limitations – physical; Social functioning; Bodily pain; Mental health; role limitation – emotional; Vitality; General health perception (PRO)
Fibromyalgia Impact Questionnaire (FIQ) [44, 45]	Health status in FM: physical functioning; work status; depression; anxiety; morning tiredness; pain; stiffness; fatigue; well-being (PRO)
Pain Intensity NRS	Pain: 0-10 NRS from “no pain” to “unbearable/worst possible pain” (PRO)
Patient Global Impression of Change (PGI-C)	Change: 7-point categorical scale rating change from the start of study from “very much worse” to “very much improved” (PRO)
Hospital Anxiety and Depression Scale (HADS) [46]	Mood: Anxiety; Depression (PRO)
Arizona Sexual Experiences Scale (ASEX) [47]	Sexual Dysfunction (PRO)
Barkley Deficits in Executive Functioning Scale-short form (BDEFS-SF) [48];	Cognitive function: executive functioning (PRO)
Multiple Ability Self-Report Questionnaire (MASQ) [49]	Cognitive function: language; visuo-perceptual; verbal memory; visual memory; attention (PRO)
Paced Auditory Serial Addition Test (PASAT) [50]	Cognitive function: auditory information processing speed and flexibility; calculation ability (administered by trained examiner)
Auditory Consonant Trigram (ACT) [51]	Working memory (administered by a trained examiner)

The following standard set of psychometric analyses was performed [16].

### Item-level evaluation

Item-level evaluation was conducted to examine data completeness, the distribution of responses per item was examined to identify any floor or ceiling effects and the pattern of missing item levels.

### Confirmatory factor analysis (CFA)

#### Initial CFA of 17-item, five-factor latent-model

**Fig. 1:**
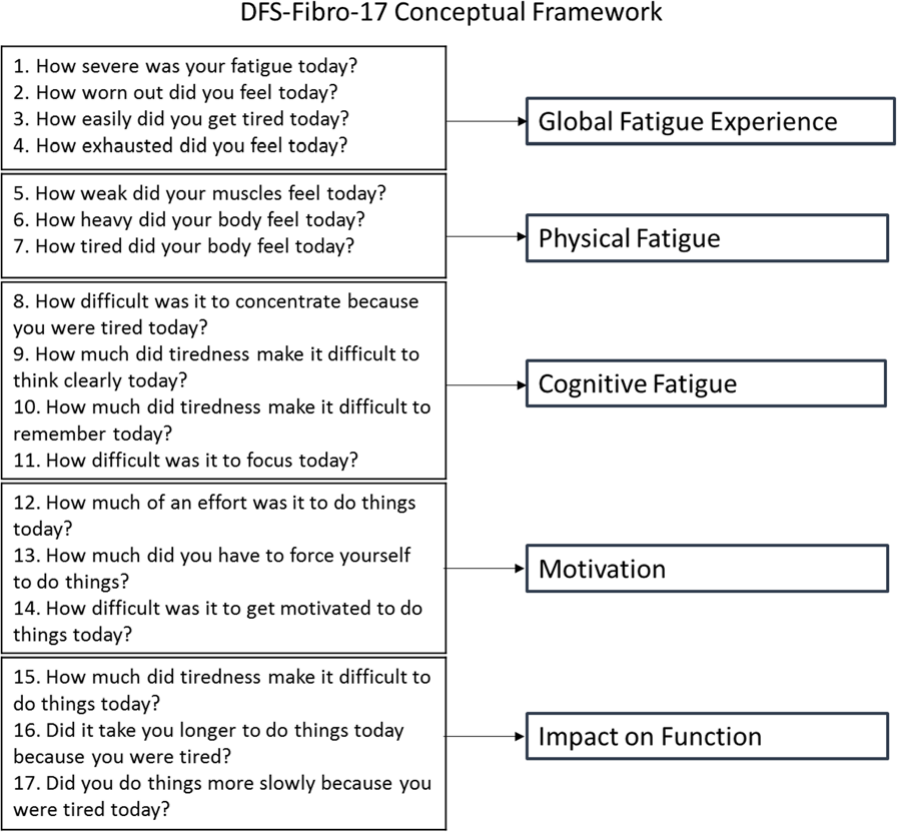
MDF-Fibro-17 Hypothesized Model

The factor structure of the MDF-Fibro-17 items was evaluated using the 17-item, five-factor latent-model (Fig. 1) analyses using interim baseline data from the Phase 2 study (*N* = 192) to assess the degree to which the hypothetical conceptual measurement model fit the data.

#### Second CFA of 5 domains to create a total score

Following the initial CFA conducted to explore the multidimensional domain structure of the measure, a secondary factor analysis of the domains was conducted to explore the appropriateness of calculating a total score (Fig. 1). This second CFA was conducted using full data set from the Phase 2 study (*N* = 381). The averaged domain raw scores were used as the manifest variables in a single-factor CFA.

For the initial and secondary CFA, the goodness of fit of the models was evaluated by several fit indices using the following pre-defined thresholds: a comparative fit index (CFI) of 0.95 or higher; a root mean square error of approximation value (RMSEA) of 0.06 or lower; a non-normed fit index (NNFI) of 0.90 or higher; and a standardized root mean residual (SRMR) of 0.08 or lower [17–23]. Confirmatory factor analysis was conducted using Mplus Version 6.1.19.

### Item-domain relationships

The relationships between individual items and the proposed MDF-Fibro-17 domains were evaluated. Item-total correlations, within the hypothesized domains, were expected to be 0.4 or greater [24–26].

### Reliability

The consistency of the items to measure fatigue at individual time points as well as the repeatability while patients were considered stable were evaluated. Reliability of the MDF-Fibro-17 domain and total scores were assessed using test-retest reliability (intra-class correlation coefficient [ICC] ≥ 0.7; Spearman Brown) and internal consistency (Cronbach’s alpha > 0.8) [18, 24]. The former was used specifically to determine the repeatability of the observed score in the absence of an observed change and the latter to assess the level of internal consistency ratings across a group of items within a domain.

### Construct (convergent and divergent) validity

Convergent validity was assessed by looking at correlations with other measures of fatigue (the MAF Global Fatigue Index [GFI] the SF-36 Vitality [VT] subscale). A moderate relationship (>0.4) was expected with overall FM severity (FIQ Total score), and measures of physical functioning on the SF-36 physical functioning (PF) subscale and physical component score (PCS).

Divergent validity was assessed by looking at correlations with measures assessing concepts other than fatigue, such as mood (HADS), sexual function (ASEX), and cognitive function (BDEFS-FS, MASQ, PASAT, and ACT) and other aspects of HRQoL measured on the remaining 6 subscales on the SF-36v2.

Moderate or greater correlations (>0.4) were expected to confirm convergent validity, and weaker correlations (<0.4) expected to confirm divergent validity. However, given the complex relationships between symptoms in FM, correlations with measures assessing concepts other than fatigue were not expected to be zero. These analyses were conducted on absolute scores at Baseline and repeated at End of Study using change scores calculated for each measure.

### Known-groups validity

Known-groups validity was examined to provide further evidence of construct validity. Scores on measures indicative of overall severity of condition (the pain intensity NRS and FIQ total score), and the GFI, a measure of fatigue, were divided into quintiles. Mean MDF-Fibro-17 total and domain scores were computed for each quintile. A generalized linear model provided an overall F-test for the group discrimination with effect size estimates considered as 0.2 (small), 0.5 (moderate), and 0.8 (large) [27].

### Sensitivity to change and responder analysis

Effect sizes are defined as the mean change found in a variable divided by the standard deviation (SD) of that variable. Effect sizes are used to translate “the before and after changes” into a standard unit of measurement that will provide a clearer understanding the relative sensitivity and performance of each clinical variable. The ability of the MDF-Fibro-17 to detect changes observed in the clinical study was evaluated using distribution- and anchor-based methods. Distribution-based methods include estimations based on observed variance in the sample such as the evaluation of ½ SD or 1 standard error of measurement. Anchor-based methods allow for the conceptual linking (e.g., discriminability) between additional known clinical or patient variables.

For the distribution-based analyses, 2 definitions for the ½ SD approach were used: ½ of the baseline SD and ½ of the change score SD; and 2 for the standard error of the mean (SEM) approach: SEM based on the ICC (test-retest coefficient and the baseline SD), and SEM based on the ICC and the change score SD [28–30].

For the anchor-based analyses, a collapsed PGI-C scale category of “very much improved” and “much improved” versus remaining PGI-C responses denoting minimal improvement, no change, or decline (“minimally worse” to “very much worse”) was used for discrimination on the MDF-Fibro-17 (see Table 1 for further details). Additional anchors of a change of 8.0 points on the GFI, and 11.0 points on the FIQ total score were also used based upon the meaningful change established for these measures [31–40].

All analyses, unless otherwise specified, were conducted using Statistical Analysis Software (SAS) software Version 9.1.3 (SAS Institute Inc., Cary, NC, US). Values reported in text are means ± SD.

## Results

### Sample characteristics

The final sample of 392 patients in the intention-to-treat (ITT) population (369 females, 23 males) had an average age of 45.7 ± 10.6 years. The majority of patients were Caucasian (82.7%) followed by Black/African American (13.0%) (Table 2). At Baseline, patients had an average FIQ total score of 54.9 ± 14.92, which indicated moderate FM severity [32]. The average pain intensity NRS score was 6.1 ± 1.31 and average GFI score was 33.4 ± 8.09. Demographic and baseline clinical characteristics of the ITT analysis group are detailed in Table 2.

**Table 2:** Demographics and Baseline Clinical Characteristics (ITT Analysis group)

Demographics	Study 0092 ITT Analysis group (*N* = 392)
Age	
Mean (SD)	45.7 (10.6)
18-45 years, n (%)	179 (45.7)
46-65 years, n (%)	213 (54.3)
Gender, n (%)	
Male	23 (5.9)
Female	369 (94.1)
Race, n (%)	
Caucasian	324 (82.7)
Black or African American	51 (13.0)
Asian	2 (0.5)
American Indian or Alaska Native	3 (0.8)
Native Hawaiian or other Pacific Islander	1 (0.3)
Multiple	9 (2.3)
Other	2 (0.5)
Duration of Fibromyalgia	
N	391
Mean (SD)	7.2 (6.77)
Median; Min, Max	5.2; 0.0, 44.0
GFI Score at Baseline	
N	384
Mean (SD)	33.4 (8.09)
Median; Min, Max	34.4; 1.0, 49.4
Baseline Pain-NRS	
N	392
Mean (SD)	6.1 (1.31)
Median; Min, Max	6.0; 3.3, 10.0
Baseline FIQ Total Score	
N	384
Mean (SD)	54.9 (14.92)
Median; Min, Max	55; 12.0, 91.7

*FIQ* Fibromyalgia Impact Questionnaire, *GFI* Global Fatigue Index from the, MAF; *ITT* intention-to-treat, *NRS* Numeric Rating Scale, *SD* standard deviation

A total of 381 (97%) patients from the ITT population had data available on the DFS-Fibro at Baseline. This analysis set was used in the psychometric evaluation of the measure.

### Item-level evaluation

The items were administered via electronic PDA, which did not allow items to be skipped; therefore, there no missing data were at the item level. No floor or ceiling effects at the item level were observed (0.3-1.3% and 0.3–0.5% respectively). All items showed a negative skew, with the majority of values to the right of the mean. Nine items had a z-score greater than 2.0 indicating a substantial departure from normality.

### Confirmatory factor analysis (CFA)

#### Initial CFA of 17-item, five-factor latent-model

An initial CFA conducted using preliminary baseline data from the TD-9855 Phase 2 study (*N* = 192) concluded that the MDF-Fibro-17 fit the data well with all parameters met the pre-specified criteria. The initial CFA model was evaluated on the 17 items, 5-factor model hypothesized for the MDF-Fibro 17 and suggest that the model fit the data from both studies. These results are presented in Table 3 below, for reference also included are initial results from the existing validation study that was reviewed to inform the development of the tool, discussed elsewhere [12].

**Table 3:** Previous Confirmatory Factor Analyses of Item-level Results

	X^2^	CFI	NNFI	RMSEA (90% CI)	SRMR
Previously Published Study (*n* = 138) [1, 9]					
MDF-Fibro Short-Form k = 17, Five-Factor Model	198.17 (df = 109)	0.96	0.95	0.077 (0.060 – 0.094)	0.016
Phase 2 Baseline Data (*n* = 192)					
MDF-Fibro k = 17, Five-Factor Model	213.43 (df = 109)	0.95	0.93	0.071 (0.056 – 0.085)	0.025

*CFI* comparative fit index, *CI* confidence interval, *NNFI* non-normed fit index, *RMSEA* root mean square error of approximation, *SRMR* standardized root mean square residual

#### Second CFA of 5 domains to create a total score (current study)

Using data collected in the full-dataset TD-9855 Phase 2 study (*N* = 381), the averaged domain raw scores were used as the manifest variables in a single-factor CFA to explore the appropriateness of a total score. The CFA models were evaluated in a stepwise fashion to allow for accumulation of evidence surrounding the dimensionality of the MDF-Fibro-17. The single-factor CFA model was evaluated on 5 domain scores and the total of 5 domain scores and suggest that the model fit the data. The CFI and NNFI were both 0.952, above their respective 0.95 and 0.90 required thresholds. The SRMR was 0.020, below the prespecified 0.08 threshold, which is, in part, due to the small number of parameters in this model. Due to the presence of correlated residuals between Fatigue Experience and the Physical Fatigue domain items, the RMSEA (0.15) was short of recommended standards and was associated with a notably high modification index (>10.0; amount of reduction if constraints removed). The path coefficients for the 5 domain MDF-Fibro-17, before accounting for correlated residuals, were 0.92 for Global Fatigue Experience, 0.88 for Cognitive Fatigue, 0.87 for Physical Fatigue, 0.98 for Motivation and 0.99 for Impact on Function.

The second-order CFA confirmed that it is acceptable to calculate a total score, which consists of all domain scores. The CFI was 0.997 and NNFI was 0.992, both well above their respective 0.95 and 0.90 required thresholds. The SRMR (0.010) was well under the required threshold, and the RMSEA (0.061) also met required standards. The path coefficients for the 17-item, 5 domain MDF-Fibro, accounting for correlated residual between Global Fatigue Experience and Physical Fatigue (0.42), were between 0.88 and 0.990. The correlation coefficients for individual items ranged from 0.92 to 0.99. The CFA results are shown in Table 4.

**Table 4:** Confirmatory Factor Analysis of Domain-level

Parameter	Threshold for Acceptability	MDF-Fibro-17
Classical Statistics		
• Complete Data	≥80%	100.0%
• Floor Range	≤9%	0.3% – 1.3%
• Ceiling Range	≤9%	0.3% – 0.5%
Preliminary CFA Second Order Model Before Accounting for Correlated Residuals		
• Path confidents		0.87 – 0.99
• X^2^ (df)		45.45 (5)
• CFI	≥0.95	0.952
• NNFI	≥0.90	0.952
• RMSEA (90% CI)	<0.06	0.15 (0.109 – 0.186)
• SRMR	<0.08	0.020
Final CFA Model Accounting for Correlated Residual between Global Fatigue Experience and Physical Domain (0.42)		
• Path confidents		0.86 – 0.99
• X^2^ (df)		9.68 (4)
• CFI	≥0.95	0.997
• NNFI	≥0.90	0.992
• RMSEA (90% CI)	<0.06	0.061 (0.007 – 0.111)
• SRMR	<0.08	0.010

*CFI* comparative fit index, *CI* confidence interval, *MDF-Fibro-17* Multidimensional Daily Diary of Fatigue-Fibromyalgia-17, *NNFI* non-normed fit index, *RMSEA* root mean square error of approximation, *SRMR* standardized root mean square residual

### Item-domain relationships

Corrected item-total correlations within hypothesized domains ranged from 0.92 to 0.96 for Global Fatigue Experience, 0.96 to 0.98 for Cognitive Fatigue, 0.85 to 0.91 for Physical Fatigue, 0.94 to 0.96 for Motivation, and 0.93 to 0.97 for Impact on Function, all of which met pre-defined criteria and were considered substantial. For all items except two, observed correlations were highest with its own domain compared to with other domains. Item “How tired did your body feel today?”, part of the Physical Fatigue domain, correlated more strongly with Global Fatigue Experience (0.92), Motivation (0.88), and Impact on Function (0.88) than its own domain (0.85). Item “How much did tiredness make it difficult to do things today?”, part of the Impact on Function domain had a slightly higher correlation with the Motivation domain than its own domain (0.95 versus 0.93). All correlations are presented in Table 5.

**Table 5:**
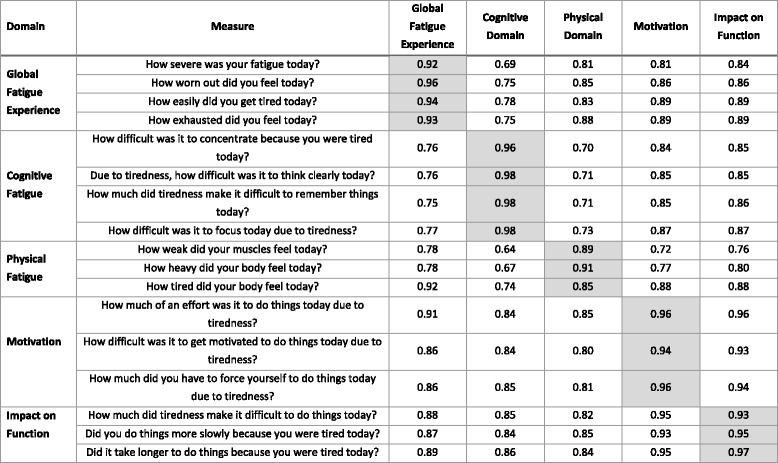
Corrected Item-Level Psychometrics: Item-Total Correlations

Highlighted correlations indicate same-scale item-total correlations

### Reliability

Test-retest reliability was assessed by evaluating the reproducibility of MDF-Fibro-17 scores over the time period between Baseline and Day 8 and from Week 5 to Week 6. All ICCs (Spearman Brown) exceeded the required 0.70 level, for baseline versus day 8, ICCs ranged from 0.71 to 0.82 (median of 0.74), and for Week 5 versus Week 6 all exceeded 0.90.

Internal consistency was confirmed as acceptable with strong Cronbach’s alpha for the total score and all domain scores (ɑ = 0.94-0.99). Reliability data are shown per MDF-Fibro domain in Table 6.

**Table 6:** Psychometric Testing of Final Questionnaire (Reliability, Construct Validity, Responsiveness)

Parameter	Threshold for Acceptability	MDF-Fibro-17					
		Total	Global Fatigue Experience	Cognitive Fatigue	Physical Fatigue	Motivation	Impact on function
Reliability [18, 24]							
• Cronbach’s Alpha	≥0.8	0.99	0.98	0.99	0.94	0.98	0.98
• Test-retest (ICC) Baseline vs. Week 1/Week 5 versus Week 6	≥0.7	0.76/0.92	0.71/0.90	0.82/0.94	0.73/0.91	0.75/0.91	0.76/0.92
Construct Validity							
• Convergent Validity at Baseline/End of Active Treatment	>0.4						
o GFI		0.73/0.80	0.75/0.84	0.62/0.69	0.64/0.75	0.73/0.77	0.72/0.76
o SF-36 VT		0.52/0.66	0.52/0.68	0.43/0.57	0.46/0.60	0.53/0.65	0.50/0.62
o FIQ Total		0.71/0.80	0.69/0.81	0.59/0.69	0.67/0.76	0.68/0.77	0.70/0.76
o SF-36 PF		0.42/0.40	0.42/0.39	0.31/0.34	0.43/0.38	0.41/0.39	0.44/0.40
o SF-36 PCS		0.41/0.50	0.41/0.50	0.28/0.40	0.43/0.50	0.41/0.49	0.43/0.49
• Divergent validity at Baseline/End of Active Treatment	< Convergent						
o Mood		0.36/0.52	0.29/0.47	0.36/0.53	0.32/0.41	0.34/0.53	0.34/0.52
o Cognitive function		0.06/0.47	0.04/0.39	0.07/0.54	0.08/0.36	0.03/0.48	0.13/0.47
o SF-36 other scales		0.27/0.66	0.26/0.66	0.26/0.56	0.27/0.65	0.26/0.59	0.26/0.63
o Sexual Function		0.18/0.21	0.15/0.17	0.18/0.23	0.14/0.17	0.19/0.22	0.19/0.21
• Known-groups Validity	*p* < 0.05	<0.001	<0.001	<0.001	<0.001	<0.001	<0.001
Responsiveness and Effect Size							
• Change from Baseline to End of Active Treatment	*p* < 0.05	<0.001	<0.001	<0.001	<0.001	<0.001	<0.001
• Effect Size	0.8 and above (Large)	0.91	0.95	0.69	0.94	0.88	0.85

Correlations with other measurements were collected as part of the Phase 2 study. The results are available upon request

*FIQ* Fibromyalgia Impact Questionnaire, *GFI* Global Fatigue Index, from the MAF, *ICC* intraclass correlation coefficient, *MDF-Fibro-17* Multidimensional Daily Diary of Fatigue-Fibromyalgia-17, *SF-36* 36-Item Short Form Health Survey, *PCS* SF-36 Physical Composite Score, *PF* physical functioning, *VT* SF-36 Vitality subscale

### Construct (convergent and divergent) validity

These data indicate overall good construct validity for the MDF-Fibro-17. Correlations with measures hypothesized to capture the same or a highly related concept, demonstrating convergent validity, were moderate (>0.4) to high (>0.7) at Baseline and End of Study for MDF-Fibro-17 scores. The highest correlations for each of the MDF-Fibro-17 total and domain scores were with the GFI (0.62 to 0.84), the FIQ Total (0.59 to 0.81), and the SF-36 VT (0.43 to 0.68). The majority of the correlations with the SF-36 measures of physical functioning – the PF and PCS – were all at least moderate with the exception of the MDF-Fibro-17 Cognitive Fatigue domain at Baseline versus PF and PCS (-0.31 and -0.28 respectively), and the MDF-Fibro-17 Global Fatigue Experience, Cognitive Fatigue, Physical Fatigue, and Motivation domains against the SF-36 PF at End of Study (-0.39, -0.34, -0.38 and -0.39 respectively). The results for convergent validity are presented in Table 6.

With respect to divergent validity, weaker correlations were observed, with low correlations (<0.4) between all MDF-Fibro-17 total and domain scores versus sexual function (ASEX) at Baseline and End of Study, and all measures of cognitive function (MASQ, PASAT, ACT, and BDEFS-SF), mood (HADS), and the other SF-36 subscales at Baseline. Low to moderate correlations were observed at the End of Study Treatment visit (Day 43; 0.36 to 0.66). The results for divergent validity are presented in Table 6.

### Known-groups validity

All known-group difference analyses of MDF-Fibro-17scores were highly significant (*p* < 0.001) when performed using quintiles. Large effect sizes (>0.8),[27, 41] determined by the F value, provided an indication of the differential sensitivity of the MDF-Fibro-17 scores to the cross-sectional known-groups, showing the greatest ability to discriminate between the 5 quintiles on the NRS, GFI, and FIQ Total. Scores by quintiles are summarized in Table 7.

**Table 7:** Scores by GFI, FIQ-Total, and Pain NRS Quintiles

MDF-Fibro-17 Scores	Q1	Q2	Q3	Q4	Q5	F Value	Probability F
Tests of Known-groups Validity by GFI Quintile							
Total	4.02	4.94	5.59	6.42	7.19	84.37	<0.001
Global Fatigue Experience	4.38	5.52	6.02	6.78	7.48	88.67	<0.001
Cognitive Fatigue	3.23	4.02	4.76	5.71	6.47	48.02	<0.001
Physical Fatigue	4.71	5.47	5.90	6.76	7.37	53.77	<0.001
Motivation	3.93	4.91	5.71	6.55	7.53	86.88	<0.001
Impact on Function	3.82	4.77	5.54	6.30	7.08	74.76	<0.001
Tests of Known-groups Validity by FIQ-Total Quintile							
Total	3.99	5.01	5.69	6.26	7.23	81.63	<0.001
Global Fatigue Experience	4.48	5.51	6.07	6.64	7.52	77.75	<0.001
Cognitive Fatigue	3.17	4.30	4.82	5.39	6.54	43.89	<0.001
Physical Fatigue	4.57	5.37	6.15	6.71	7.44	67.05	<0.001
Motivation	3.93	5.06	5.80	6.41	7.45	73.08	<0.001
Impact on Function	3.80	4.82	5.61	6.14	7.18	77.70	<0.001
Tests of Known-groups Validity by Pain NRS Quintiles							
Total	4.35	4.97	5.57	6.33	7.13	53.58	<0.001
Global Fatigue Experience	4.69	5.38	5.97	6.75	7.58	72.11	<0.001
Cognitive Fatigue	3.65	4.20	4.80	5.52	6.18	23.90	<0.001
Physical Fatigue	4.74	5.41	6.00	6.61	7.65	64.24	<0.001
Motivation	4.44	5.06	5.64	6.51	7.20	39.74	<0.001
Impact on Function	4.22	4.80	5.42	6.26	7.03	47.41	<0.001

*FIQ* Fibromyalgia Impact Questionnaire, *GFI* Global Fatigue Index from the MAF, *MDF-Fibro-17* Multidimensional Daily Diary of Fatigue-Fibromyalgia-17, *NRS* Numeric Rating Scale, *Q* quintile

### Sensitivity to change and responder analysis

Significant (*p* < 0.001) changes were observed in all MDF-Fibro-17 scores from Baseline to End of Study. A medium effect size (>0.5) was observed for the Cognitive Fatigue domain (-0.69). Effect sizes for the total score and all other domains were large (-0.85 to -0.95). Similar effect sizes to those observed on the MDF-Fibro-17 were also observed in the pain intensity NRS, FIQ total score, GFI, and SF-36 VT.

The responder definitions for the MDF-Fibro-17 domains were assessed using distribution and anchor-based approaches. Similar results were found with both distribution-based approaches, used to understand the lower limits of acceptable responder definitions. Anchor-based responder definitions using the PGIC ([Patients’ Global Impression of Change] very much/much improved category), GFI (>11-point improvement), and FIQ total score (>8-point improvement) were similar to those determined by selected distribution based methods (-2.55 to -2.94). However, the responder definitions determined using the PGIC much improved category, GFI, and FIQ had a broader range (-2.06 to -3.41). The mean responder score, based on the anchor-based analyses, for the MDF-Fibro-17 Total Score and the 5 domains ranged from -2.48 to -2.85. Overall, the recommended responder cut-off for the total score as well as the other domains is -2.5 (summarized in Table 8).

**Table 8:** Responder Analysis Results

	MDF-Fibro-17 Scores					
Definition	Total	Global Fatigue Experience	Cognitive Fatigue	Physical Fatigue	Motivation	Impact on Function
Distribution-based Methods						
½ Baseline SD (< -0.50 SD)	-2.55	-2.57	-2.65	-2.54	-2.87	-2.72
½ Δ Score SD (< -0.50 SD)	-2.57	-2.63	-2.59	-2.64	-2.94	-2.75
SEM: ICC and baseline SD (< -1 SEM)	-2.55	-2.58	-2.56	-2.57	-2.87	-2.70
Anchor-based Methods						
PGIC Very Much/Much Improved	-2.39	-2.41	-2.14	-2.39	-2.59	-2.42
Change from Baseline in GFI < -11	-3.17	-3.23	-2.86	-3.15	-3.41	-3.17
Change from Baseline in FIQ Total < -8	-2.30	-2.32	-2.06	-2.33	-2.44	-2.32
Overall						
Mean	-2.59	-2.62	-2.48	-2.60	-2.85	-2.68
Median	-2.55	-2.58	-2.58	-2.56	-2.87	-2.71

∆ change score, *FIQ* Fibromyalgia Impact Questionnaire, *GFI* Global Fatigue Index from MAF, *ICC* intraclass correlation, *LS* least squares, *MDF-Fibro-17* Multidimensional Daily Diary of Fatigue-Fibromyalgia-17, *Pain-NRS* Pain Numerical Rating Scale, *PGIC* Patient Global Impression of Change, *SD* standard deviation, *SEM* standard error of measurement, *SF-36* Medical Outcomes Study 36-Item Short-Form Health Survey

## Discussion

The MDF-Fibro-17 is a multidimensional measure of FM-related fatigue, made up of 5 domains (Global Fatigue Experience, Cognitive Fatigue, Physical Fatigue, Motivation, and Impact on Function). The analyses confirmed the domain structure suggested by the conceptual model developed from in-depth qualitative work with FM patients, and indicated sound psychometric properties of the measure.

All 17 items in the MDF-Fibro-17 performed well as individual items and as part of the 5 domain structure of the instrument. The multidimensional structure allows the MDF-Fibro-17 to capture the broad experience of FM-related fatigue, a characteristic that has been identified as important within the clinical and regulatory community [1, 7, 8, 10]. In addition, the factor analyses confirmed that it is also appropriate to calculate a single total score informed by the in-depth measurement of FM-related fatigue. The relationships between individual items within and across domains demonstrates the complexity of fatigue in FM. There was a strong correlation observed between motivation and physical functioning items in particular, suggesting potential item redundancy. However, both the qualitative data and conceptual model [9] highlighted that these are related but distinct aspects of FM-related fatigue from the patient perspective and therefore relevant and important to include within the measure.

Tests of internal consistency and test-retest reliability were strong, indicating that this is a highly reliable measure. The correlations observed between the MDF-Fibro-17 and other measures in the study hypothesized to be either similar (convergent validity) or dissimilar (divergent validity) were overall as expected, confirming good construct validity. The strongest relationships were observed between the MDF-Fibro-17 and overall FM severity (FIQ Total) and the GFI, another measure of fatigue. The moderate correlations with the SF-36 VT, a single item evaluating a simple concept similar to fatigue, and some of the measures for divergent validity demonstrate the high level of complexity of FM-related fatigue, in which multiple symptoms are experienced and, though distinct, are closely related.

Known-groups analysis revealed that the MDF-Fibro-17 total and domain scores were able to differentiate between all groups tested. Highly significant changes were observed over the study period on all scores of the MDF-Fibro-17, with medium to large effect sizes, which reflected the changes observed on other outcomes in the study, indicate that the instrument is sensitive to detecting changes observed in a clinical study.

Responder analyses conducted using different definitions for both anchor based and distribution-based techniques produced similar estimates and the results suggested a reasonable responder cut-off to be around -2.5.

One limitation to this study is that although the MDF-Fibro-17 has the potential to assess the different components of FM-related fatigue based on data described above, this study was conducted in a particular clinical trial population in response to drug therapy intervention. Therefore, responsiveness and sensitivity to other therapies would need to be further explored in future studies.

## Conclusion

The psychometric evaluation and strong evidence of content validity indicate that the MDF-Fibro-17 is a relevant, psychometrically robust, multidimensional instrument, with sensitivity to detection change and clear response definitions. Taken as a whole, the MDF-Fibro-17 has the potential to become a reliable clinical outcome assessment tool to evaluate fatigue in adult patients with FM within a clinical trial setting [12].

## Abbreviations

ACT: Auditory consonant trigram
ASEX: Arizona sexual experiences scale
BDEFS-SF: Barkley deficits in executive functioning scale – short form
CFA: Confirmatory factor analysis
CFI: Comparative fit index
FDA: Food and drug administration
FIQ: Fibromyalgia impact questionnaire
FM: Fibromyalgia
GFI: Global fatigue index
HADS: Hospital anxiety and depression scale
HRQoL: Health-related quality of life
ICC: Intraclass correlation coefficient
ITT: Intention-to-treat
MAF: Multidimensional assessment of fatigue
MASQ: Multiple ability self-report questionnaire
MDF-Fibro-17: Multidimensional daily diary of fatigue-fibromyalgia-17
NNFI: Non-normed fit index
NRS: Numeric rating scale
PASAT: Paced auditory serial addition test
PCS: Physical component score
PDA: Personal digital assistant
PF: Physical functioning
PGIC: Patients’ global impression of change
PRO: Patient reported outcome
RMSEA: Root mean square error of approximation
SAS: Statistical analysis software
SD: Standard deviation
SEM: Standard error of the mean
SF-36: 36-Item short form health survey
SRMR: Standardized root mean square residual
US: United States
VT: Vitality

## References

[1] Humphrey L, Arbuckle R, Mease P, Williams DA, Samsoe BD, Gilbert C (2010). Fatigue in fibromyalgia: a conceptual model informed by patient interviews. BMC Musculoskelet Disord.

[2] Lawrence RC, Felson DT, Helmick CG, Arnold LM, Choi H, Deyo RA (2008). Estimates of the prevalence of arthritis and other rheumatic conditions in the United States. Part II Arthritis Rheum.

[3] Wolfe F, Smythe HA, Yunus MB, Bennett RM, Bombardier C, Goldenberg DL (1990). The American college of rheumatology 1990 criteria for the classification of fibromyalgia. Report of the multicenter criteria committee. Arthritis Rheum.

[4] Wolfe F, Ross K, Anderson J, Russell IJ, Hebert L (1995). The prevalence and characteristics of fibromyalgia in the general population. Arthritis Rheum.

[5] Wolfe F, Clauw DJ, Fitzcharles MA, Goldenberg DL, Katz RS, Mease P (2010). The American college of rheumatology preliminary diagnostic criteria for fibromyalgia and measurement of symptom severity. Arthritis Care Res (Hoboken).

[6] Perrot S, Winkelmann A, Dukes E, Xu X, Schaefer C, Ryan K (2010). Characteristics of patients with fibromyalgia in France and Germany. Int J Clin Pract.

[7] Schaefer C, Chandran A, Hufstader M, Baik R, McNett M, Goldenberg D (2011). The comparative burden of mild, moderate and severe fibromyalgia: results from a cross-sectional survey in the United States. Health Qual Life Outcomes.

[8] Mease PJ, Arnold LM, Crofford LJ, Williams DA, Russell IJ, Humphrey L (2008). Identifying the clinical domains of fibromyalgia: contributions from clinician and patient Delphi exercises. Arthritis Rheum.

[9] Burbridge C, Symonds T, Humphrey L, Arbuckle R, Hirsch I, Whelan L. Validation of the Daily Diary of Fatigue Symptoms-Fibromyalgia (DFS-Fibro). Open J Rheumatol Autoimmune Disord. 2013;3(2):92–103.

[10] Arnold LM, Wang F, Ahl J, Gaynor PJ, Wohlreich MM (2011). Improvement in multiple dimensions of fatigue in patients with fibromyalgia treated with duloxetine: secondary analysis of a randomized, placebo-controlled trial. Arthritis Res Ther.

[11] Mease PJ, Palmer RH, Wang Y (2014). Effects of milnacipran on the multidimensional aspects of fatigue and the relationship of fatigue to pain and function: pooled analysis of 3 fibromyalgia trials. J Clin Rheumatol.

[12] Morris S, Burbridge C, Symonds T, Hudgens S. Multidimensional Daily Diary of Fatigue Symptoms Fibromyalgia-17 Items (MDF-Fibro-17). Part 1: Development and Content Validity. 2015. doi:10.1186/s12891-017-1544-y. 10.1186/s12891-017-1544-y PMC5434627 28511678

[13] Guidance for industry: patient-reported outcome measures: use in medical product development to support labeling claims. 2009. U.S. Food and Drug Administration. https://www.fda.gov/downloads/drugs/guidances/ucm193282.pdf. 10.1186/1477-7525-4-79 PMC1629006 17034633

[14] Smith JA, Patil DL, Daniels OT, Ding YS, Gallezot JD, Henry S (2015). Preclinical to clinical translation of CNS transporter occupancy of TD-9855, a novel norepinephrine and serotonin reuptake inhibitor. Int J Neuropsychopharmacol.

[15] Theravance Technical Report for the Psychometric Study of the Multidimensional Daily Diary of Fatigue Symptoms-Fibromyalgia (DFS-Fibro-17). Version 2.0. 9-22-2014. San Diego, California, Covance Market Access Services Inc. (internal report, unpublished).

[16] Cappelleri JC, Zou KH, Bushmakin AG, Alvir JMJ, Alemayehu D, Symonds T (2013). Patient-reported outcomes: measurement, implementation and interpretation.

[17] Bentler PM (1990). Comparative fit indexes in structural models. Psychol Bull.

[18] Cronbach LJ (1951). Coefficient alpha and the internal structure of tests. Psychometrika.

[19] Hu L, Bentler PM (1998). Fit indices in covariance structure modeling: sensitivity to underparameterized model misspecification. Psychol Methods.

[20] Hu L, Bentler PM (1999). Cutoff criteria for fit indexes in covariance structure analysis: conventional criteria versus new alternatives. Struct Equ Model.

[21] Marsh HW, Hau KT, Wen Z (2004). In search of golden rules: Comment on hypothesis-testing approaches to setting cutoff values for fit indexes and dangers in overgeneralizing Hu and Bentler’s (1999) findings. Struct Equ Model.

[22] Steiger JH, Lind JC (1980). Statistically based tests for the number of common factors.

[23] Steiger JH (1990). Structural model evaluation and modification: an interval estimation approach. Multivar Behav Res.

[24] Nunnally JC (1978). Psychometric theory.

[25] Comrey AL, Lee HB (1992). A first course in factor analysis.

[26] McHorney CA, Ware JE, Lu JF, Sherbourne CD (1994). The MOS 36-item short-form health survey (SF-36): III. Tests of data quality, scaling assumptions, and reliability across diverse patient groups. Med Care.

[27] Cohen J. Statistical power analysis for the behavioral sciences rev. Hillsdale: Lawrence Erlbaum Associates, Inc; 1977.

[28] Fayers P, Machin D. Quality of life: the assessment, analysis and interpretation of patient-reported outcomes. West Sussex: John Wiley & Sons; 2013.

[29] Litwin MS. How to measure survey reliability and validity, 7 edn. Thousand Oaks: Sage Publications; 1995.

[30] Vaz S, Falkmer T, Passmore AE, Parsons R, Andreou P (2013). The case for using the repeatability coefficient when calculating test-retest reliability. PLoS One.

[31] Arnold LM, Gendreau RM, Palmer RH, Gendreau JF, Wang Y (2010). Efficacy and safety of milnacipran 100 mg/day in patients with fibromyalgia: Results of a randomized, double-blind, placebo-controlled trial. Arthritis Rheumatism.

[32] Bennett RM, Bushmakin AG, Cappelleri JC, Zlateva G, Sadosky AB (2009). Minimal clinically important difference in the fibromyalgia impact questionnaire. J Rheumatol.

[33] Branco JC, Zachrisson O, Perrot S, Mainguy Y (2010). A European multicenter randomized double-blind placebo-controlled monotherapy clinical trial of milnacipran in treatment of fibromyalgia. J Rheumatol.

[34] Geisser ME, Palmer RH, Gendreau RM, Wang Y, Clauw DJ (2011). A pooled analysis of Two randomized, double-blind, placebo-controlled trials of milnacipran monotherapy in the treatment of fibromyalgia. Pain Pract.

[35] Mease PJ, Clauw DJ, Gendreau RM, Rao SG, Kranzler J, Chen W (2009). The efficacy and safety of milnacipran for treatment of fibromyalgia. a randomized, double-blind, placebo-controlled trial. J Rheumatol.

[36] Oh TH, Hoskin TL, Luedtke CA, Weingarten TN, Vincent A, Kim CH (2012). Predictors of clinical outcome in fibromyalgia after a brief interdisciplinary fibromyalgia treatment program: single center experience. PM&R.

[37] Ohta H, Oka H, Usui C, Ohkura M, Suzuki M, Nishioka K (2012). A randomized, double-blind, multicenter, placebo-controlled phase III trial to evaluate the efficacy and safety of pregabalin in Japanese patients with fibromyalgia. Arthritis Res Ther.

[38] Pauer L, Atkinson G, Murphy TK, Petersel D, Zeiher B (2012). Long-term maintenance of response across multiple fibromyalgia symptom domains in a randomized withdrawal study of pregabalin. Clin J Pain.

[39] Pouchot J, Kherani RB, Brant R, Lacaille D, Lehman AJ, Ensworth S (2008). Determination of the minimal clinically important difference for seven fatigue measures in rheumatoid arthritis. J Clin Epidemiol.

[40] Russell IJ, Perkins AT, Michalek JE (2009). Sodium oxybate relieves pain and improves function in fibromyalgia syndrome: a randomized, doubleGÇÉblind, placeboGÇÉcontrolled, multicenter clinical trial. Arthritis Rheumatism.

[41] Hedges LV, Olkin I (1985). Statistical methods for meta-analysis.

[42] Belza BL, Henke CJ, Yelin EH, Epstein WV, Gilliss CL (1993). Correlates of fatigue in older adults with rheumatoid arthritis. Nurs Res.

[43] Ware JE, Kosinski M, Dewey JE. How to score version 2 of the SF-36 health survey (standard & acute forms). Lincoln: QualityMetric Incorporated; 2000.

[44] Burckhardt CS, Clark SR, Bennett RM (1991). The fibromyalgia impact questionnaire: development and validation. J Rheumatol.

[45] Bennett RM, Friend R, Jones KD, Ward R, Han BK, Ross RL (2009). The revised fibromyalgia impact questionnaire (FIQR): validation and psychometric properties. Arthritis Res Ther.

[46] Zigmond AS, Snaith RP (1983). The hospital anxiety and depression scale. Acta Psychiatr Scand.

[47] McGahuey A (2000). The Arizona sexual experience scale (ASEX): reliability and validity. J Sex Marital Ther.

[48] Allee-Smith PJ, Winters RR, Drake A, Joslin AK. Test Review: Barkley Deficits in Executive Functioning Scale (BDEFS). J Psychoeduc Assess. 2012;31(1):80–83.

[49] Seidenberg M, Haltiner A, Taylor MA, Hermann BB, Wyler A (1994). Development and validation of a multiple ability self-report questionnaire. J Clin Exp Neuropsychol.

[50] Gronwall DMA (1977). Paced auditory serial-addition task: a measure of recovery from concussion. Percept Mot Skills.

[51] Brown J (1958). Some tests of the decay theory of immediate memory. Q J Exp Psychol.

